# Supplementary material for: An RNA Virome Analysis of the Pink-Winged Grasshopper Atractomorpha sinensis
Source: Insects. 2022 Dec 22;14(1):9. doi: 10.3390/insects14010009 (PMC9862791; doi:10.3390/insects14010009)
Supplement: Supplementary file 1 [file insects-14-00009-s001.zip › Supplementary Table S1.pdf]

Supplementary Table S1. Primers used for RT-PCR in this study

Primers used for RT-PCR	Forward primer (5'-3')	Reverse primer (5'-3')
**Atractomorpha sinensis nege-like virus 1 (ASNLV1)**		
ASNLV1 5′-RACE GSP	TGTTAGGAACCGCATAACCTCCGCGATACGT	CTAATACGACTCACTATAGGGCAAGCAGTGGTATC AACGCAGAGT
ASNLV1 3′-RACE GSP	ATGATGAGGATGGAATTGTCACCGTCAG	CTAATACGACTCACTATAGGGC
ASNLV1 segment1	GCGATCATAAGGCTCAGAATTGTAGTA	TGACACTAGTTGTTAATTCTTCCACTCT
ASNLV1 segment2	GGGATTATACTAGGAACGGTTTTAAGTGT	TCTGTAATAAACTATGGAGCGGTCGACT
ASNLV1 segment3	CAGCTTGGCGTGATAACGGTTATGTTG	ACACATAAAGTGGCAGACATACCCAACATAG
**Atractomorpha sinensis iflavirus 1 (ASIV1)**		
ASIV1 5′-RACE GSP	GGAATAATACTCTCTGTCAATAAATTGACTC	CTAATACGACTCACTATAGGGCAAGCAGTGGTATC AACGCAGAGT
ASIV1 3′-RACE GSP	GAATGGACAGCTATGTATGGAATGCCTGAG	CTAATACGACTCACTATAGGGC
ASIV1 segment1	GTGGATCCTGGCCTTGCCCC	AACTGCTCCGCTAACACATCGC
ASIV1 segment2	TCGCGATGTGTTAGCGGAGCA	GGACCGGACCCCGTTTCGTC
**Atractomorpha sinensis ollusvirus 1 (ASOV1)**		
ASOV1 segment1	TTTAAAACAATCAAACACAACAAC	GTTGTTGTGTTTGATTGTTTTAAA

ASOV1 segment2	CTGATCTGCTCAGGACCCTCAGTT	ATTAATGACTTCTGGAAGCGGATGGT
ASOV1 segment3	TTGCCACATCATCAGCATAGGCAGGA	CTAGCTTAGAAATTCACCTGAGCTT
**Atractomorpha sinensis chu-like virus 1 (ASCLV1)**		
ASCLV1 segment1	TTATAATTCATTGTTGTTCATACAT	GTATAAATGTAAGAAATGTTTCACA
ASCLV1 segment2	AACAAATTTAGAGCTTGAAAC	AGGGGACTGGATTTCCAGGGCA
**Aphid lethal paralysis virus (ALPV)**		
ALPV segment1	CCCGGATATTTGCGCCCGTTTACTG	CCTGCGCCGCTATGGGTTTTGGTGC
ALPV segment2	GACGTTATATTGGATGCACCAAACTG	CGCGCTACATGAGCTATAGTCATCT
ALPV segment3	GAGGTATAAATTTTCACTTTAAA	CTCAAAGAAGAAAAGTGAGAAAAACTT
ALPV segment4	ATGTCAGGTACTTTTTCAACAACGCTCCCC	GCTCTCAATCAATTTTCCATAAGCTGGTGA
