# Supplementary material for: An RNA Virome Analysis of the Pink-Winged Grasshopper Atractomorpha sinensis
Source: Insects. 2022 Dec 22;14(1):9. doi: 10.3390/insects14010009 (PMC9862791; doi:10.3390/insects14010009)
Supplement: Supplementary file 1 [file insects-14-00009-s001.zip › Supplementary Table S2.pdf]

Supplementary Table S2. Abbreviations of virus names and GenBank accession numbers used in this study

Abbreviations	Complete virus name	Accession (genome)	Accession (RdRp)
Nege-like virus			
INGV	Ingleside virus	MW741891.1	AYP67558.1
GDNLV1	Ganwon-do negev-like virus 1	MT757507.1	QPN36965.1
NECV	Nephila clavipes virus 4	NC_040614.1	YP_009552461.1
HVLV13	Hubei virga-like virus 13	KX883803.1	APG77733.1
Kitaviridae			
HGSVC2	Hibiscus green spot virus 2	NC_016141.1	NC_016141.1
CILVC2	Citrus leprosis virus C2	NC_038848.1	JX000024.1
HICV	Hibiscus-infecting cilevirus	MW413437.1	ATW76030.1
Nelorpivirus			
NEV	Negev virus	NC_030294.1	AFI24684.1
MAV	Manglie virus	MH807827.1	QBR99594.1
NELV	Negev-like virus	NC_035129.1	CCV01575.2
NGV	Ngewotan virus	MH745161.1	QDC23193.1
CAV	Castlerea virus	NC_034569.1	AQZ55393.1
NVD1	Nelorpivirus dungfly 1	MT344120.1	MT344120
Sandewavirus			
SVD1	Sandewavirus dungfly 1	MT344121.1	MT344121
AHAENV	Andrena haemorrhoa nege-like virus	NC_040813.1	YP_009553581.1
TANAV	Tanay virus	NC_024071.1	AYU75416.1
BUSV	Bustos virus	MN101546.1	BAU71147.1
SANV	Santana virus	JQ675606.1	AFI24675.1

UXMV	Uxmal virus	MH719095.1	AYW01743.1
BVM11	Biggievirus Mos11	KX924639.1	ASO75598.1
Chuviridae			
LSV1	Lishi spider virus 1	KM817597.1	AJG39051.1
HCLV3	Hubei chuvirus-like virus 3	NC_033015.1	YP_009337089.1
Dicistroviridae			
Aparavirus			
ABPV	Acute bee paralysis virus	NC_002548.1	AAG13118.1
KBV	Kashmir bee virus	NC_004807.1	NP_851403.1
IAPV	Israeli acute paralysis virus	NC_009025.1	AYE20099.1
SIV1	Solenopsis invicta virus 1	NC_006559.1	ADI46702.1
Cripavirus			
EPV	Empeyrat virus	KU754505.1	AMO03208.1
DCV	Drosophila c virus	NC_001834.1	NP_044945.1
CPV	Cricket paralysis virus	NC_003924.1	BCZ95743.1
RPV	Rhopalosiphum padi virus	NC_001874.1	QWO79067.1
ALPV	Aphid lethal paralysis virus	NC_004365.1	AIJ00043.1
Iflaviridae			
Iflavirus			
STIFV3	Soybean thrips iflavirus 3	MT195548.1	QQN90112.1
SBPV	Slow bee paralysis virus	NC_014137.1	ABS84820.1
ARIFV	Armigeres iflavirus	NC_036585.1	YP_009448183.1
YIFV1	Yongsan iflavirus 1	MW699047.1	BBQ04785.1
PBV13	Png bee virus 13	MT482495.1	QKW94218.1
BUBV6	Bundaberg bee virus 6	MG995707.1	AWK77862.1
DWV	Deformed wing virus	NC_004830.2	UDY80777.1

AIFV1	Apis iflavirus 1	MZ822075.1	UCR92483.1
LDIFV1	Lymantria dispar iflavirus 1	NC_024497.1	AIF75200.1
HELFV	Heliconius erato iflavirus	NC_024016.1	YP_009026409.1
HCV1	Hubei coleoptera virus 1	NC_033030.1	YP_009337127.1
TCIFV	Tribolium castaneum iflavirus	MG012488.1	AUE23905.1
Virgaviridae			
Furovirus			
CWMV	Chinese wheat mosaic virus	NC_002359.1	NP_059513.1
Hordeivirus			
BSMV	Barley stripe mosaic virus	NC_003469.1	AHY22372.1
Chuviridae			
Scarabeuvirus			
LSV1	Lishi spider virus 1	KM817597.1	AJG39051.1
HCLV3	Hubei chuvirus-like virus 3	NC_033015.1	YP_009337089.1
LNCLV1	Lampyris noctiluca chuvirus-like virus 1	MH620818.1	QBP37027.1
WCV3	Wuchang Cockroach Virus 3	NC_043472.1	YP_009666256.1
HCLV1	Hubei chuvirus-like virus 1	NC_033327.1	APG78716.1
Culicidavirus			
CMV5	Culex mosquito virus 5	MH188036.1	AXQ04841.1
CMV4	Culex mosquito virus 4	MH188031.1	QRW42867.1
IRV1	Imjin River virus 1	NC_028482.1	YP_009182177.1
WMV8	Wuhan Mosquito Virus 8	NC_028265.1	YP_009177719.1
Mivirus			
HM3	Hebei mivirus 3	MZ244270.1	QYW06809.1
LM	Lesnoe mivirus	MN542369.1	QPD01622.1
KTV	Karukera tick virus	MN599998.1	QGW51122.1

HM1	Hebei mivirus 1	MZ244262.1	QYW06785.1
XJM1	Xinjiang mivirus 1	MZ244269.1	QYW06807.1
unclassified			
BCRV148	Blattodean chu-related virus OKIAV148	MT153417.1	QMP82200.1
Piscichuvirus			
SASV	Sanxia atyid shrimp virus	KX884439.1	APG78770.1
WFCLV	Wenling fish chu-like virus	MG600010.1	AVM87275.1
HFV1	Herr Frank virus 1	MN567051.1	QHX39772.1
GRBSCLV	Guangdong red-banded snake chuvirus-like virus	MG600009.1	AVM87272.1
Aliusviridae			
Ollusvirus			
CUV	Culverton virus	MN167499.1	QED21529.1
SRBV	Scaldis River bee virus	KY053857.1	UDL14020.1
HCV3	Hubei coleoptera virus 3	NC_032922.1	YP_009336866.1
TLV	Taiyuan leafhopper virus	MH708020.1	AYN64867.1
SFV1	Shayang Fly Virus 1	NC_031214.1	YP_009300663.1
ACLV5	Atrato Chu-like virus 5	MN661033.1	QHA33675.1
Iflaviridae			
Iflavirus			
SAV	Sacbrood virus	NC_002066.1	QGY64205.1
SBPV	Slow bee paralysis virus	NC_014137.1	YP_003622540.1
