# Supplementary material for: An RNA Virome Analysis of the Pink-Winged Grasshopper Atractomorpha sinensis
Source: Insects. 2022 Dec 22;14(1):9. doi: 10.3390/insects14010009 (PMC9862791; doi:10.3390/insects14010009)
Supplement: Supplementary file 1 [file insects-14-00009-s001.zip › Supplementary Table S3.pdf]

Supplementary Table S3. Amino acid/nucleotide identity analysis of ASNV1 based on the conserved amino acid and nucleotide sequence of the RdRp domain

	Identity	1	2	3	4	5	6	7	8	9	10	11	12	13	14	15	16	17	18	19	20	21	22	23
**Sandewavirus**	SDV1	***	**73.5**	**31**	**60.1**	**59.4**	**54.7**	**57.8**	**56.7**	**28**	**30.2**	**27.5**	**33.6**	**34.3**	**33.8**	**34.5**	**35.2**	**12.8**	**10.3**	**28.7**	**29.9**	**30**	**30.5**	**26.2**
	AHNV	69.2	***	**31.4**	**62.2**	**58.7**	**52.3**	**55.5**	**56**	**28.8**	**30.4**	**28.4**	**33.3**	**34.2**	**33.7**	**34**	**35.4**	**13**	**11**	**26.4**	**29.6**	**28.2**	**29.5**	**27.3**
	NDV1	42.7	43.2	***	**31.2**	**31.1**	**29.7**	**32**	**31.7**	**35.3**	**37.8**	**35.5**	**61.9**	**61.6**	**61.2**	**61.2**	**61.2**	**10.8**	**10.2**	**34.7**	**35.9**	**35.6**	**33.3**	**31**
	TANAV	61.9	60.6	40.6	***	**66.7**	**57.7**	**64.6**	**64**	**27.8**	**29.6**	**27.8**	**31.5**	**32.2**	**32.2**	**32.2**	**32.6**	**14.1**	**11.3**	**26.4**	**28.9**	**30.5**	**29.6**	**26.7**
	BUSV	63	59.9	40	63.3	***	**61.3**	**70.1**	**65.8**	**25.4**	**28**	**25.7**	**31.5**	**32.2**	**32.5**	**32.7**	**32.9**	**12.3**	**10.5**	**28**	**28.1**	**31.8**	**29.4**	**24.6**
	SANV	58.7	55.9	42.1	60.8	61	***	**59.4**	**59.3**	**27.4**	**29.9**	**28.1**	**31.6**	**32.1**	**32.5**	**32.3**	**32.8**	**11.4**	**9.5**	**29**	**28.6**	**29.5**	**27.3**	**24.8**
	UXMV	59.3	59.1	41.9	62.7	67.1	62.2	***	**65.6**	**26.4**	**29.5**	**26.6**	**32.2**	**32.7**	**32.5**	**33.4**	**33.2**	**12.6**	**9.4**	**26.7**	**27.6**	**30.1**	**28.5**	**24.6**
	BVM11	59.4	60.6	42.9	61.8	62.6	59.1	63.5	***	**27.3**	**29.5**	**28.1**	**31.4**	**32.6**	**32.9**	**32.9**	**33.3**	**12.4**	**11.2**	**29.3**	**29.6**	**32.3**	**30.4**	**26.3**
**Kitaviridae**	CILVC2	39.7	39.4	46.1	39.2	38.9	40.9	40.1	40.4	***	**51.7**	**95.7**	**35.3**	**36.6**	**35.3**	**36.9**	**36.2**	**11.2**	**8.9**	**33.1**	**33.9**	**35**	**35.3**	**28.4**
	HGSVC2	40.9	40	47.3	40.5	39.5	40.6	39.5	39.8	55.7	***	**52.4**	**38.1**	**38.6**	**37.6**	**38.6**	**39**	**12.2**	**11**	**35.6**	**34.6**	**34.9**	**34.3**	**29.1**
	HICV	41.2	39	47.4	40	39.3	41.8	39.4	42.4	85.2	57.1	***	**35.7**	**36.9**	**35.7**	**36.9**	**36.4**	**11.4**	**9.6**	**34.1**	**33.9**	**35.2**	**35**	**28.9**
**Nelorpivirus**	CAV	41.8	42.8	60.8	38.8	40.2	41.3	41.5	42.4	46.9	48.6	47.4	***	**82.6**	**83.5**	**82.4**	**80.8**	**11.3**	**11.1**	**33.3**	**37.5**	**35.4**	**34.2**	**31.2**
	NEV	40.5	43.1	60.1	39.5	40	41.3	40.1	40.7	46.6	49	48.1	72.2	***	**85.6**	**97.5**	**86.5**	**9.7**	**10.8**	**35.7**	**37.3**	**35.6**	**34.9**	**31**
	MAV	41.5	41.6	59.7	39.4	38.6	41.4	40	41.2	47.9	47.2	47.4	71.6	73.6	***	**86.2**	**91.9**	**10.2**	**10.4**	**35.2**	**37.5**	**34.9**	**34.5**	**30.5**
	NELV	59	59.1	43.2	62.5	64.4	60	64.3	85.5	40.9	38.9	41.2	42.3	40.7	41	***	**86.2**	**10.2**	**10.8**	**35.5**	**37**	**35.1**	**34.9**	**30.5**
	NGV	41.2	42.4	60	39.7	39	40.8	40.6	43.5	47.3	49.7	48	71.3	74	83.3	41.7	***	**10.4**	**11.3**	**36**	**37**	**36.3**	**35.4**	**31**
**Chuviridae**	LSV1	35.2	36	34	33.3	34.5	34.3	34	34.4	33	31.6	33.5	31.2	30.2	31.9	36.4	31.5	***	**48.6**	**12.6**	**12.7**	**13.8**	**12**	**11.8**
	HCLV3	34.7	35.3	34.1	33.6	33.7	34.1	34.5	34.4	32.8	31.8	32.9	31	32.1	32.5	35.7	32.6	57.5	***	**11.7**	**13**	**13.8**	**13.2**	**8.9**
**Nege-like virus**	INGV	31.1	30.3	31	31.4	31.8	30.9	32.6	31.6	29.8	29.6	31.7	31.9	31.5	31.5	31.8	32	29.7	29.2	***	**53.5**	**52**	**50.8**	**44.1**
	GDNLV1	42	40.2	48.7	39.7	37.8	39.6	39.4	38.6	46	44.9	45.7	47.1	50	48.1	39.5	48.6	34.7	33.8	30.3	***	**59.4**	**54.5**	**45.9**
	NECV	44.7	42.4	50	41.9	41.6	40.1	40.7	43.2	46.8	47.9	47.2	45.7	45.7	47.1	44.2	46	34.3	33	27.9	59.3	***	**77**	**44.3**
	HVLV13	43.2	41.9	48.6	41.3	40.6	39.5	40.1	40.6	46	47	46.6	45.5	45.3	46.3	41.4	45.6	33.4	32.8	29.9	57.9	73.5	***	**42.9**
	**ASNV1**	40.1	39	47.1	40.2	38.3	40.3	38.8	38.4	45	44.3	45.1	44.9	41.7	44.3	40.3	43.7	33.1	31.2	28.2	52.4	54.8	53	***

Bold text indicates amino acid identity. Non-bold text indicates nucleotide identity. The numbers 1-23 represent virus from in the left column. Virus names and GeneBank accessions numbers are listed in Supplementary Table S2.
