# Supplementary material for: An RNA Virome Analysis of the Pink-Winged Grasshopper Atractomorpha sinensis
Source: Insects. 2022 Dec 22;14(1):9. doi: 10.3390/insects14010009 (PMC9862791; doi:10.3390/insects14010009)
Supplement: Supplementary file 1 [file insects-14-00009-s001.zip › Supplementary Table S4.pdf]

Supplementary Table S4. Amino acid/nucleotide identity analysis of ASIV1 based on the conserved amino acid and nucleotide sequence of the RdRp domain

		Identity	1	2	3	4	5	6	7	8	9	10	11	12	13	14	15	16	17	18	19	20	21	22	23	24
**Dicistroviridae**	**Aparavirus**	ABPV	***	86.1	87.1	63.7	43.1	46.5	49.5	38.1	39.4	32.1	29.3	28.7	29.8	34.5	30.5	31.2	31.5	32.3	32.7	32.5	33.1	30.1	8.5	7.4
		KBV	75.8	***	93.2	63.8	40.8	44.6	47.9	36.8	37.4	30.2	27.4	27.2	28.3	33	29.7	30.8	31	33.3	32.3	31.6	31.9	29.3	7.2	6.2
		IAPV	77.6	85.7	***	63.4	40.9	46.5	50.2	38.5	39.1	30.1	28	28.2	28.6	33.2	30.1	30.4	32.1	33.5	33	33.2	32.6	29.6	8.5	7.4
		SIV1	61.6	61.3	62.4	***	43.3	49.2	50.8	35.8	36.7	32.5	30.1	28.4	28.8	33.5	33.5	32.2	32.5	33	34.3	38	36.6	35.7	9.4	7.3
	**Cripavirus**	EPV	54	53.6	52.5	54.3	***	46.8	53.5	34.5	36.7	27.9	27.4	22	23.7	30.8	28.3	21.8	22.1	27.2	26.8	31.2	22.7	21.9	7.6	10.5
		DCV	54.3	54.2	53.2	55.4	57.7	***	71.9	40.1	39.3	27.6	28.4	30	29.3	32.5	28.8	30.2	31.2	32.5	32.6	32.3	30.3	29.7	8.2	8.3
		CPV	52.3	53.6	54.7	54.3	58.8	67.6	***	40.1	37.3	29	28.8	29.3	29.3	31	29.2	32	31.8	33.2	32.6	31.9	30.3	31	8.6	10.7
		RPV	47.8	46.9	46.6	48.1	48.6	50.8	49.6	***	53.7	29.9	28.7	28.5	28.8	29.5	30.4	32.5	31.7	33.5	33.2	33.2	32	28.9	7.6	7.3
		ALPV	50.8	48.6	50.8	45.8	48.3	50.2	48.6	57.9	***	31.5	30.3	30.4	30.1	34.4	32.4	32.1	31	34.8	34.9	34.8	34.9	30.1	7.8	5.9
**Iflaviridae**	**Iflavirus**	STIFV3	46.3	44.8	45.4	44.2	44.3	45.3	43.8	42.9	42	***	53.8	42.1	44.4	43.3	42.9	44.8	40.9	43.7	47	42.6	46	42.9	9.8	9.2
		SBPV	44.3	41.8	44.5	43.3	42.1	44.8	41.8	41.3	42.1	54.4	***	37.9	38.2	42.2	41.9	42.1	39.5	41.9	41.2	41.1	40.8	37.5	8.5	8.5
		ARIFV	45.5	43.8	45.8	44.6	42.7	45.3	42.7	43.7	45.2	50.8	50.8	***	72.5	38.8	40.3	35.4	33.1	37.8	35.3	36.2	33.7	32.8	9	8
		YIFV1	43.6	43.1	46.2	44.2	41.2	44.6	42.6	43.1	43.2	51.1	50.1	74.4	***	40.2	40.3	35.8	34.6	38.1	38.1	38.7	36.8	32.9	9.5	7.8
		PBV13	44.7	45	45.3	44	46.1	45.1	42.9	43.8	44.9	50.3	48.8	49.4	48.8	***	64.4	63.8	55	48.6	52	50.4	50.2	44	10.1	8.7
		BUBV6	45.8	44.3	46.4	45.6	44.6	44.2	43.5	43.6	43	51.2	48.9	48.9	48.1	61.4	***	83.9	56.2	48.9	50.9	49.8	49.1	44.1	10.1	10.4
		DWV	44.2	46.8	47	43.6	46.6	45.5	45.1	44.3	43.7	53	48.7	49.3	49.5	62.4	71.9	***	51.2	48.6	51.8	49.5	43.2	40.8	8.5	8.7
		AIFV1	45.9	44.2	44.8	46.3	44.8	47	45.2	42.3	43.3	50.7	47.9	48.1	49.4	56.8	59.5	56.9	***	48	48	47.6	46.5	42.9	9.4	7.7
		LDIFV1	45.2	47.5	48.8	48.1	45.1	46.6	44.4	45.5	47.7	53.6	53	48.9	49.9	55.1	53.7	53.9	55.3	***	77.7	50.9	51.3	44	8.4	8
		HELFV	45.5	45.3	46.8	46.7	44.8	47.9	44.5	43.5	46.4	54.1	52	50.6	49.6	56.5	52.5	56.3	56.2	73.7	***	51.8	51.6	44.8	8.7	9.5
		HCV1	46.9	46.1	45.9	49	46.3	45.3	43.8	44.8	44.9	52	50.1	49.9	50.2	55.5	55	55.3	56.3	58.2	56.7	***	62.7	51.4	9.2	9.5
		TCIFV	36.7	41.4	39.7	40.7	41.2	40.2	38.9	36.7	35.7	37	36	34.4	29.9	38.6	34.4	36.7	35.5	38.3	37	36	***	41.3	9.4	8.9
		**ASIV1**	47	45.8	47.3	47.1	46	42.6	43.4	40.3	42.4	53.8	49.7	50.7	49.6	51	51.5	53.2	49.1	53.7	53.3	59.4	34.4	***	9.3	9.8
**Virgaviridae**	**Furovirus**	CWMV	32.4	35	34.1	33.4	32.3	32.9	33.4	31.5	32.3	32.8	34.2	34.9	34.9	32.1	33.5	31.9	34.1	33.5	33.6	34.8	26.9	32.6	***	56.8
	**Hordeivirus**	BSMV	32.1	34.2	34	34.4	34	34.8	32.3	31.9	32.5	34.5	34.6	34.4	34.1	34.3	33.1	31.5	34.7	34.7	36.5	35.9	27.8	33.7	58.8	***

Bold text indicates amino acid identity. Non-bold text indicates nucleotide identity. The numbers 1-24 represent virus from in the left column. Virus names and GeneBank accessions numbers are listed in Supplementary Table S2.
