# Supplementary material for: An RNA Virome Analysis of the Pink-Winged Grasshopper Atractomorpha sinensis
Source: Insects. 2022 Dec 22;14(1):9. doi: 10.3390/insects14010009 (PMC9862791; doi:10.3390/insects14010009)
Supplement: Supplementary file 1 [file insects-14-00009-s001.zip › Supplementary Table S5.pdf]

Supplementary Table S5. Amino acid/nucleotide identity analyses of ASCV1 and ASOV1 based on the conserved amino acid and nucleotide sequence of the RdRp domain

			1	2	3	4	5	6	7	8	9	10	11	12	13	14	15	16	17	18	19	20	21	22	23	24	25	26	27	28	29
Chuviridae	*Scarabeuvirus*	LSV1	***	46.4	47.7	46.4	46.1	52.1	44.7	48.1	51.8	50.7	44	40.9	39.3	40.1	39.4	43	30.1	26.5	26.5	28.9	29.2	27.9	27.9	34.4	37.2	39.3	38.5	10.1	8.4
		WCV3	55.2	***	46.3	43.6	44.5	47.2	41.4	45.5	48	44.6	43.4	41	38	39.9	37.9	41.9	26.6	23.8	23.8	26.5	26.5	24.7	24.7	34.4	37.2	38.2	38.3	11.9	10.2
		HCLV3	55.3	55.1	***	52.5	45.8	49.5	42.4	47.2	50.3	48.7	46.1	42.2	39.4	43	40	44.1	27.8	25.7	25.8	28	27.4	26.2	26.2	34.4	39.5	39.7	38.9	11.7	10.4
		LNCLV1	57.5	55.3	61.6	***	45	49.9	42.5	47.3	51.8	48.7	43.1	39.4	36.7	41.3	39.4	43.1	27.4	25.7	25.7	27.8	27.2	27.2	27.2	35	38.6	37.8	37.7	11.9	9.8
		HCLV1	56.4	54.4	54.2	54.8	***	48.7	42.7	46.6	49	48.9	43.4	42.2	39.9	43.1	39.4	44	26.8	24.2	24.3	27.6	27.6	26.1	26.1	37.3	39	40.9	40.3	10.7	8.8
	Culicidavirus	CMV5	54.5	52.3	53.5	52.9	52.3	***	98.9	91.2	91.4	46.9	48.2	43.8	42.7	43.2	43.4	42.9	25.7	26.4	26.4	26.5	25.6	25.6	25.6	39.4	40.3	38.9	40.3	11.5	10.4
		CMV4	54.7	52.5	53.4	52.8	51.9	98.3	***	90.4	91.7	46.1	45	41.4	39.5	41.5	39.1	42.3	24.4	22.9	22.9	25.2	26	24.6	24.6	36.8	38	39.5	37.1	11.5	10.4
		IRV1	54.9	52.9	54.3	53.8	53.7	75.3	75.7	***	94.2	47	45.6	42.1	40.8	41.8	42.1	43.1	25.8	23.9	23.9	27	26.1	24	24	38.5	38.8	39.9	40.1	11.5	10.8
		WMV8	54.6	51.8	53	53.4	54.1	77.3	75.8	76.6	***	46.6	48.6	44.2	42.6	43.1	44.3	43.8	25.3	26.2	26.2	26.7	25.4	24.5	24.5	40.4	41.2	39.3	40.7	12.1	10.6
	unclassified	**ASCV1**	52.8	52	52.4	50.3	51	51.5	52.2	51.1	51.1	***	64.2	45.8	44.9	43.7	44.2	43.8	30.9	30.8	30.8	28.8	28.1	27.6	27.6	37.4	41.3	40.3	39	10	9
		BCRV148	51.9	52	52.2	51.1	50.6	51.4	51.2	53	52.4	61.3	***	45.2	41.8	42.2	42.7	44.5	32.9	26.5	26.5	29.7	30.3	28.7	28.7	38.6	39.8	41.5	41.4	11.4	9.8
	Mivirus	HM3	47.6	47.4	49.6	49	48	50.3	50.6	49.8	49	51.6	51	***	62.8	58.6	58.8	59.5	28.5	25	25	26.2	27	26.8	26.8	36.7	38.5	40	38.7	13.1	9.4
		LM	48.4	49.2	47.7	48.6	48.8	48.9	49.1	50.1	50.4	51.4	48.8	62.8	***	57.7	55.2	57.3	28.3	23.7	23.7	26.2	27.4	26.6	26.6	34.3	34.5	36.8	35.7	11.9	9.8
		KTV	48.4	48.4	51.2	51.9	48.6	50.6	51	50.5	49.3	52.1	51.3	58.8	59	***	86	85.9	28	24.8	24.8	26.9	27.3	26.7	26.7	36.7	38.5	38.3	36.7	11.9	9
		HM1	48.5	49.4	49.4	52	48.2	50.7	50.8	51	49.2	51.8	50.7	59	58.4	73.6	***	98.8	29.7	24.9	24.9	27.2	28.4	27.2	27.2	34.2	37.9	38.3	37.4	12.3	8.8
		XJM1	49.4	49.5	50.1	51.3	48.1	50.4	50.7	50.9	49	51.9	51.7	59.7	58.4	73.5	97.1	***	29.6	26.1	26.1	26.9	28.4	27.1	27.1	36.8	38.6	38.2	37.3	12.1	8.6
Aliusviridae	*Ollusvirus*	ACLV5	43.9	40.3	40.1	42.2	42.9	39.5	39.5	39.9	41.1	42.1	43.2	39.8	40.2	38.6	40.6	40.1	***	42.8	42.8	42	44	43.2	43.2	25.8	28.5	26.9	27.2	9.5	8.8
		TLV	40.5	37.9	40.1	40	39.7	39	38.9	39.2	39.5	40.7	39.8	36.9	38.5	39.1	38.6	38.6	48.8	***	100	47.9	47.7	46	46	25.6	25.3	26	26.7	9.8	8.4
		SFV1	39.7	38.7	39.3	40.4	41.4	38	37.4	38	39	39.7	39.8	37.8	38.4	37.5	37.9	37.5	52.4	49.7	***	48	47.8	46	46	25.6	25.3	26	26.7	9.8	8.4
		HCV3	42.2	41	40.5	41.2	42.8	39	38.5	38.5	41	39.5	39.4	38.9	38.8	40.3	39.2	39	53.5	51.1	54.9	***	49.5	50.8	50.8	27.7	28.5	26.5	26	10.3	9.2
		**ASOV1**	39.9	38	38.6	39.2	37.6	39.4	39	39	38.9	39.4	39.9	38.8	39.1	39.9	39.2	39.4	51	51.5	51.1	52.7	***	50.1	50.1	26.1	26.6	26	27.4	9.6	7.8
		CUV	31.5	28.8	29.8	30.5	31.4	30.7	30.8	29.9	31.2	28.7	29.7	29.5	28.1	28.9	30	29.4	29.5	29.5	28.6	30.2	28.5	***	100	25.3	27.6	27.9	27.1	10.4	8.2
		SRBV	41.9	40	42	41.8	42.6	39.7	39.1	38.7	40.8	39.6	40	37	38.6	38.3	38.8	38.5	51.7	51	55.3	56.2	52.2	29	***	25.3	27.6	27.9	27.1	10.4	8.2
Chuviridae	*Piscichuvirus*	SASV	47.7	48.3	46.8	48.9	51.5	48.4	48.8	49	49.7	47.5	46.9	45.5	44.9	45.7	45.1	45	40.6	41	39.8	41.1	38.7	31.2	40.1	***	49.7	52.6	50.1	9.6	8.6
		HFV1	33.7	34.5	31.7	33.7	35.7	32.1	32.3	32.5	31.8	31.2	32.8	31.7	31.3	30.1	31.2	30.9	32.7	32.6	31.7	33.7	32.4	40.7	32.3	34.6	***	62.7	63.9	11.4	7.4
		WFCLV	46.7	47.6	48.6	48.5	48.8	47.1	47.5	48.5	47.7	46	48.7	47.3	45.5	46.5	48.3	48.3	38.4	38.1	39.2	37.9	37	29.4	38.9	54.3	31.3	***	62.3	11.6	10
		GRBSCLV	48.5	48.2	47.4	48.2	51	47	47.6	49.6	48.6	46.1	49.4	45.1	44.9	45.7	46.4	46.5	40	40.2	39.9	39.7	38.2	29.1	40.4	55.9	32.6	61.8	***	11.2	9.2
Iflaviridae	*Iflavirus*	SAV	34.9	32.3	32.9	33.8	34	33.5	33.3	32	34	29.3	34.1	33.7	34.2	32.2	31.2	31.6	32	31.4	31.1	32.1	29.9	27.3	32.8	33	32.2	31.7	32.1	***	25.6
		SBPV	35.2	33.4	31.9	33.1	36.3	33	33.3	31.5	33.3	31.9	33.4	31.8	32.9	32.3	33.6	32.9	33.6	32.5	31.5	34.8	31.8	29.4	33.4	33.4	33.8	31.6	32.8	51	***

Bold text indicates amino acid identity. Non-bold text indicates nucleotide identity. The numbers 1-29 represent virus from in the left column. Virus names and GeneBank accessions numbers are listed in Supplementary Table S2.
