# Supplementary figures and images for: An RNA Virome Analysis of the Pink-Winged Grasshopper Atractomorpha sinensis
Source: Insects. 2022 Dec 22;14(1):9. doi: 10.3390/insects14010009 (PMC9862791; doi:10.3390/insects14010009)

Figure S2. *Atractomorpha sinensis* chu-like virus 1

**A**

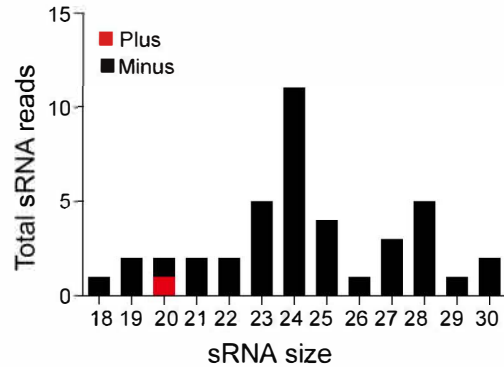

**B**

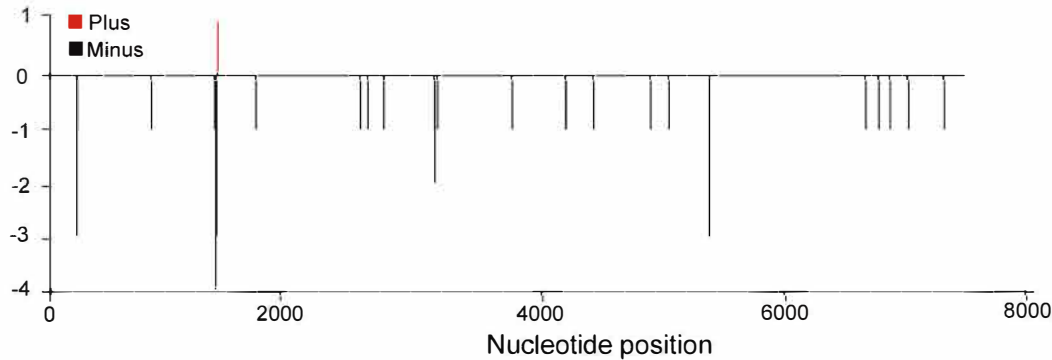

Supplement: Supplementary file 1 [file insects-14-00009-s001.zip › Supplementary Figure S2.pdf]
